# Shivering: scores and protocols

**DOI:** 10.1186/cc11267

**Published:** 2012-06-07

**Authors:** Neeraj Badjatia

**Affiliations:** 1 Neurological Institute of New York, Columbia University College of Physicians and Surgeons, New York, USA

## 

Shivering is both an anticipated consequence and, potentially, a major adverse effect of therapeutic hypothermia. Even mild hypothermia can elicit a vigorous thermoregulatory defense to maintain body temperature at the hypothalamic set point. In healthy humans, peripheral vasoconstriction is triggered at 36.5°C and shivering at 35.5°C. Temperature thresholds for vasoconstriction and shivering are often higher than normal in brain-injured patients; therefore, these thermoregulatory defenses may occur more vigorously and at higher temperatures in these individuals. Control of shivering is essential for effective cooling, as shivering fights the cooling process, makes attaining target temperature difficult, is extremely uncomfortable, and can trigger massive increases in systemic and cerebral energy consumption and metabolic demand. The first step in treatment is adequate tools to recognize shivering. The Bedside Shivering Assessment Scale is a simple, validated four-point scale that enables repeated quantification of shivering at the bedside. Therapy for shivering should ideally stop or suppress the central thermoregulatory reflex rather than just uncoupling this response from skeletal muscle contraction, as the latter approach does not mitigate the ongoing cerebral and systemic stress response. Analgo-sedation with opioids, α_2_-receptor agonists, or propofol is almost always effective as a last resort to prevent shivering. However, nonpharmacological strategies as first-line interventions for shivering minimize the risk of excessive sedation, which can make neurological examination difficult and increase the risk of complications. The Columbia Anti Shivering protocol has been developed with these strategies in mind, and we base our approach on prospectively collected cooling data on 213 patients who underwent 1,388 patient-days of temperature modulation. Eighty-nine patients underwent hypothermia and 124 patients underwent induced normothermia. In 18% of patients and 33% of the total patient-days, only none-sedating baseline interventions were needed. The first agent used was most commonly dexmeditomidine half the time, followed by opiates and increased doses of propofol. Younger patients, men, and lower body surface area were factors associated with increased number of anti-shivering interventions. As noted by this protocol, a significant proportion of patients undergoing temperature modulation can be effectively treated for shivering without oversedation and paralysis. Patients at higher risk for needing more interventions are younger men with decreased body surface area.
